# Serum Liberation of Fetal Fibronectin Variants in Patients with Pulmonary Hypertension: ED-A^+^ Fn as Promising Novel Biomarker of Pulmonary Vascular and Right Ventricular Myocardial Remodeling

**DOI:** 10.3390/jcm10122559

**Published:** 2021-06-09

**Authors:** Laura Bäz, Michelle Roßberg, Katja Grün, Daniel Kretzschmar, Alexander Berndt, P. Christian Schulze, Christian Jung, Marcus Franz

**Affiliations:** 1 Department of Internal Medicine I, Jena University Hospital, 07740 Jena, Germany; Laura.Baez@med.uni-jena.de (L.B.); Michelle.Rossberg@uni-jena.de (M.R.); Katja.Gruen@med.uni-jena.de (K.G.); Daniel.Kretzschmar@med.uni-jena.de (D.K.); Christian.Schulze@med.uni-jena.de (P.C.S.); 2 Section of Pathology, Institute of Forensic Medicine, Jena University Hospital, 07743 Jena, Germany; Alexander.Berndt@med.uni-jena.de; 3 Department of Internal Medicine, Division of Cardiology, University Hospital Düsseldorf, Heinrich Heine University Düsseldorf, 40225 Düsseldorf, Germany; christian.jung@med.uni-duesseldorf.de

**Keywords:** fetal fibronectin variants, pulmonary hypertension, pulmonary vascular remodelling, right ventricular myocardial remodelling

## Abstract

Background and Aims: Pulmonary Hypertension (PH) represents an aetiologically and clinically heterogeneous disorder accompanied by a severely impaired prognosis. Key steps of PH pathogenesis are vascular and right ventricular myocardial remodelling entailing the re-occurrence of fetal variants of the cell adhesion modulating protein fibronectin (Fn) being virtually absent in healthy adult tissues. These variants are liberated into circulation and are therefore qualified as excellent novel serum biomarkers. Moreover, these molecules might serve as promising therapeutic targets. The current study was aimed at quantifying the serum levels of two functionally important fetal Fn variants (ED-A^+^ and ED-B^+^ Fn) in patients suffering from PH due to different aetiologies compared to healthy controls. Methods: Serum levels of ED-A^+^ and ED-B^+^ Fn were quantified using novel ELISA protocols established and validated in our group in 80 PH patients and 40 controls. Results were analysed with respect to clinical, laboratory, echocardiographic and functional parameters. Results: Serum levels of ED-A^+^ Fn (*p* = 0.001) but not ED-B^+^ Fn (*p* = 0.722) were significantly increased in PH patients compared to healthy controls. Thus, the following analyses were performed only for ED-A^+^ Fn. When dividing PH patients into different aetiological groups according to current ESC guidelines, the increase in ED-A^+^ Fn in PH patients compared to controls remained significant for group 1 (*p* = 0.032), 2 (*p* = 0.007) and 3 (*p* = 0.001) but not for group 4 (*p* = 0.156). Correlation analysis revealed a significant relation between ED-A^+^ Fn and brain natriuretic peptide (BNP) (*r* = 0.310; *p* = 0.002), six minutes’ walk test (*r* = −0.275; *p* = 0.02) and systolic pulmonary artery pressure (PAPsys) (*r* = 0.364; *p* < 0.001). By logistic regression analysis (backward elimination WALD) including a variety of potentially relevant patients’ characteristics, only chronic kidney disease (CKD) (OR: 8.866; CI: 1.779–44.187; *p* = 0.008), C reactive protein (CRP) (OR: 1.194; CI: 1.011–1.410; *p* = 0.037) and ED-A^+^ Fn (OR: 1.045; CI: 1.011–1.080; *p* = 0.009) could be identified as independent predictors of the presence of PH. Conclusions: Against the background of our results, ED-A^+^ Fn could serve as a promising novel biomarker of PH with potential value for initial diagnosis and aetiological differentiation. Moreover, it might contribute to more precise risk stratification of PH patients. Beyond that, the future role of ED-A^+^ Fn as a therapeutic target has to be evaluated in further studies.

## 1. Introduction

The term Pulmonary Hypertension (PH) summarizes a clinical and aetiological heterogeneous disease group and is defined by a mean pulmonary artery pressure (PAP) of ≥ 20mmHg measured by right heart catheterization [1]. Among the five clinical groups of PH implemented in current guidelines, group 1 (pulmonary arterial hypertension, PAH) is an orphan disease, whereas group 2 (PH due to left heart disease), group 3 (PH due to chronic lung disease and/or hypoxia) and group 4 (chronic thromboembolic PH, CTEPH) are the most frequently occurring forms showing high prevalence rates notably in the ageing society. Group 5 sums up various disorders associated with an increased PAP and can be perceived as the PH of mixed pathogenesis not matching any other clinical PH group [2]. PH per se and in particular when being accompanied by consecutive right heart failure due to pressure overload is associated with a poor prognosis [3,4,5]. With respect to therapeutic management, one has to consider that a specific treatment aimed to reduce PAP and thereby right ventricular pressure overload is recommended only for PH group 1 and group 4, e.g., by administration of dual endothelin receptor antagonists or phosphodiesterase type 5 inhibitors. For PH group 4, patients should be additionally evaluated for their eligibility for surgical pulmonary endarterectomy or balloon pulmonary angioplasty, which is a promising therapeutic strategy in selected individuals. In contrast, treatment of PH groups 2, 3 and 5 is largely restricted to the therapy of the underlying disease, e.g., left heart failure [2,6,7,8]. However, in selected patients suffering from PH group 2 and showing a pronounced pre-capillary component, a specific therapy might be beneficial after individual evaluation in specialized centres [6]. Taking these considerations into account, there are certain unmet clinical needs. First, the identification of novel circulating biomarkers could facilitate diagnosis and prognosis estimation, especially by predicting the future development of right heart failure within the PH disease continuum. Second, promising molecular targets enabling a specific treatment for the majority of PH patients, in particular PH groups 2 and 3, are long-needed and could relevantly reduce disease burden and mortality [9,10].

The development and progression of PH are accompanied by certain structural and functional alterations pertaining to both, the pulmonary vasculature with vasoconstriction, microthrombus formation and vascular remodelling and the consecutively affected right ventricle in terms of myocardial remodelling [4,11,12]. On the histopathological level, these remodelling processes are very similar at least in PH groups 1, 2 and 3 [13,14,15] and are concordantly accompanied by a reorganization of the extracellular matrix (ECM) entailing the re-occurrence of so-called fetal molecular variants of important cell adhesion modulation proteins such as tenascin-C (Tn-C) or fibronectin (Fn). These variants, generated by alternative splicing of the pre-mRNA leading to the inclusion or omission of certain extra-domains (ED), are of crucial impact during embryonic development, are nearly absent in healthy adult organs and become re-expressed in a variety of cardiovascular diseases [16,17,18,19]. Thus, they qualify as excellent biomarkers or even therapeutic targets to modify cardiovascular tissue remodelling processes, e.g., in terms of antibody-based treatment strategies [17,20,21]. Fetal variants of cellular Fn mainly occur as ED A (ED-A^+^ Fn) or ED B (ED-B^+^ Fn) containing isoforms [22]. For both, a re-occurrence in processes of pathological tissue remodelling, e.g., neoplastic diseases, chronic inflammation or cardiovascular disorders, has been described mainly in human tissue samples or preclinical disease models [23,24,25,26,27,28,29,30]. In rat and mouse models of PH, a relevant re-expression of ED-A^+^ Fn associated with both pulmonary vascular as well as right ventricular myocardial remodelling could be recently demonstrated by our group [31,32]. With respect to the potential value of ED-A^+^ or ED-B^+^ Fn as circulating biomarkers to improve diagnosis, prognosis estimation or therapy surveillance in cardiovascular diseases, there are only very limited data [33] and, up to now, there are no studies focusing on their potential role in human PH.

Therefore, the current study was aimed at quantifying the serum levels of ED-A^+^ and ED-B^+^ Fn in patients suffering from PH belonging to different clinical groups compared to healthy controls. Results will be correlated, among others, to clinical, functional, laboratory and echocardiographic parameters to analyse their potential value as novel biomarkers in PH.

## 2. Material and Methods

### 2.1. Study Population

In the present study, 80 patients suffering from pulmonary hypertension (PH) of different aetiological groups according to current guidelines [2] were prospectively included in this single-centre study in the frame of the clinical registry ‘Pulmonary Hypertension’ recently established at the Department of Internal Medicine I of the University Hospital Jena, Friedrich Schiller University Jena. A group of 40 patients at increased cardiovascular risk but without relevant cardiovascular disorders served as a control collective. The study was approved by the local ethics committee of the Medical Faculty of the Friedrich Schiller University Jena (registration number: 4732-03/16) and conducted in adherence to the current versions of the Declaration of Helsinki and good clinical practice (GCP) guidelines. All patients, as well as all control subjects, gave written informed consent for participation before inclusion in the registry.

Subjects were eligible for the control group if coronary artery disease was excluded by invasive coronary angiography. Moreover, heart failure, a relevant structural or valvular heart disease, as well as an elevated systolic pulmonary artery pressure, had to be ruled out by transthoracic echocardiography (TTE). Further exclusion criteria were: an active malignant or autoimmune disease, hyperthyroidism, infections, a history of pulmonary embolism or stroke, peripheral artery disease, the intake of oral or inhaled corticosteroids and medication with immunosuppressive agents.

In all patients and controls, detailed clinical, laboratory, functional and imaging analysis was performed when appropriate according to current guidelines and local standard operating procedures. Among others, TTE was performed to determine the structure and function of the left heart, to detect relevant valve abnormalities and, in particular, to describe right heart morphology and function by the assessment of established surrogate parameters, e.g., tricuspid annular plane systolic excursion (TAPSE), the area of the right atrium (RA), and the systolic pulmonary arterial pressure (PAPsys). In addition, the 6-minute walk distance (SMWD) was documented for each patient according to standard protocols. The baseline characteristics of both the patient group and the control collective are given in Table 1.

### 2.2. Blood Samples and Laboratory Analyses

In conjunction with clinically indicated laboratory diagnostics, blood samples were withdrawn under controlled venous stasis by puncture of a cubital vein and subjected to both routine laboratory analyses carried out according to local standard operating procedures as well as a quantification of the serum concentration of ED-A^+^ Fn and ED-B^+^ Fn using ELISA technique as described below. Therefore, collection tubes were centrifuged within 20 min, and serum was transferred into special low binding tubes (Protein LoBind, Eppendorf AG, Hamburg, Germany) and stored at −80 °C after snap freezing in liquid nitrogen to reduce artificial protein degradation. To fulfil quality standards, repeated freeze-thaw cycles were strictly avoided.

### 2.3. Serum Quantification of Fetal Variants of Fibronectin by ELISA Technique

Since there are no commercially available ELISA kits for the quantification of circulating levels of ED-A^+^ Fn and ED-B^+^ Fn, a protocol recently developed and validated in our group was used. In summary, coating of the ELISA plates (96-well plate, high binding, F-Bottom ELISA Microplate, Greiner Bio-One GmbH, Frickenhausen, Germany) was performed at room temperature overnight applying 300 µL 0.5% gelatine in PBS in each well. In between the following steps, rinsing procedures using 300 µL PBS/0.1% Tween were performed in triplicate. Serum samples (50 µL, diluted in PBS, range 1:2 to 1:8) were added and allowed to incubate for 60 min. Thereafter, bound ED-A^+^ Fn or ED-B^+^ Fn was detected by adding 100 µL antibody per well for 60 min: IST-9 for the specific detection of ED-A^+^ Fn or C6 for the specific detection of ED-B^+^ Fn (1.0 µg/mL, Sirius Biotec S.r.l., Genoa, Italy) [34,35]. In the next step, 100 µL of a highly absorbed biotinylated donkey-anti-mouse antibody (Dianova, Hamburg, Germany) was applied and allowed to incubate for 60 min. In a final step, 100 µL of streptavidin labelled with horseradish-peroxidase (HRP) (1.5 µg/mL Dianova, Hamburg, Germany) was added for 40 min. Further detection steps were carried out in adherence to standard protocols and have recently been described in detail by our group [33].

### 2.4. Statistical Analyses

Statistical analyses were performed by using IBM SPSS statistic software, version 25.0 (IBM SPSS Statistics for Windows. Armonk, NY, USA). All data are expressed as mean/median ± standard deviation (SD). The Mann–Whitney U test was used to test for significant differences in the results of quantitative ED-A^+^ Fn or ED-B^+^ Fn measurement between the different groups. Bivariate correlations of parametric variables were identified by the Spearman rank correlation test. A *p*-value ≤ 0.05 was regarded statistically significant. To identify independent predictors of PH, a multivariate analysis (stepwise multiple regression) was applied using a binary logistic model (backward elimination method: Wald). The presence of PH was defined as a dependent variable. As covariates (*n* = 10), age, diabetes mellitus (DM), coronary artery disease (CAD), atrial fibrillation (Afib), chronic kidney disease (CKD), C-reactive protein (CRP), statin therapy, chronic obstructive pulmonary disease (COPD), arterial hypertension (AH) and ED-A^+^ Fn serum levels were included into the model. Then, multistep backward elimination (removal threshold *p* > 0.10) of independent variables was performed. Finally, *p* values ≤.05 were considered to be statistically significant.

## 3. Results

### 3.1. Clinical Characterization of the Study Population

In this prospective single-centre study, 80 patients (mean age 71 ± 13 years, 61% female) suffering from PH were compared to 40 control subjects (mean age 66 ± 7 years, 67% female) at increased cardiovascular risk but without relevant structural or functional cardiac or vascular disorders. Clinical baseline characteristics, including comorbidities, medication and relevant laboratory parameters, are given in Table 1. The majority of listed comorbidities, e.g., CAD, Afib, CKD or DM, showed significantly higher incidences in the PH compared to the control group (*p* < 0.05). With respect to medication, the intake of diuretics, statins, prednisolone and inhaled corticosteroids was more frequent in the PH group (*p* < 0.05), but there were no significant differences concerning the regular ingestion of aspirin, betablockers or angiotensin-converting enzyme inhibitors/sartans (*p* = n.s.). The latter is of great importance since especially these drugs might have direct influences on cardiovascular remodelling and could thereby bias serum biomarker studies. Routine laboratory tests revealed significantly higher values for BNP, CRP and creatinine and lower values for LDL cholesterol and haemoglobin in the PH compared to the control group (*p* < 0.05).

Patients in the PH group (*n* = 80) were assigned to different aetiological groups according to the current guidelines of the European Society of Cardiology published in 2015: 13 patients (16.3%) to group 1; 30 patients (37.5%) to group 2; 11 patients (13.8%) to group 3, 12 patients (15%) to group 4 and 14 patients (17.5%) to group 2/3 (combined aetiology). The baseline clinical characteristics, including comorbidities, medication and relevant laboratory parameters in comparison between the PH groups, are given in Table 2. Most notably, between the different PH groups, there were significant differences in age, the majority of comorbidities as well as a few medications, e.g., the intake of betablockers and laboratory parameters, e.g., BNP (*p* < 0.05).

The hemodynamic parameters assessed by right heart catheterization for the different PH groups as well as the subgroups of PH group 1 are given in Table 3. Echocardiographic parameters in comparison between the PH groups are listed in Table 4. Briefly summarized, there were significant differences between PH patients and controls for PAPsys, RA area, RVEDd as well as LVEF (*p* < 0.001 for all parameters). When comparing the different clinical groups of the PH patients, only LVEF showed a trend of difference, mainly driven by the decreased values in the PH group 2 and group 2/3 (*p* = 0.050).

### 3.2. Serum Levels of ED-A^+^ Fn and ED-B^+^ Fn in PH Patients Compared to Controls

**Figure 1:**
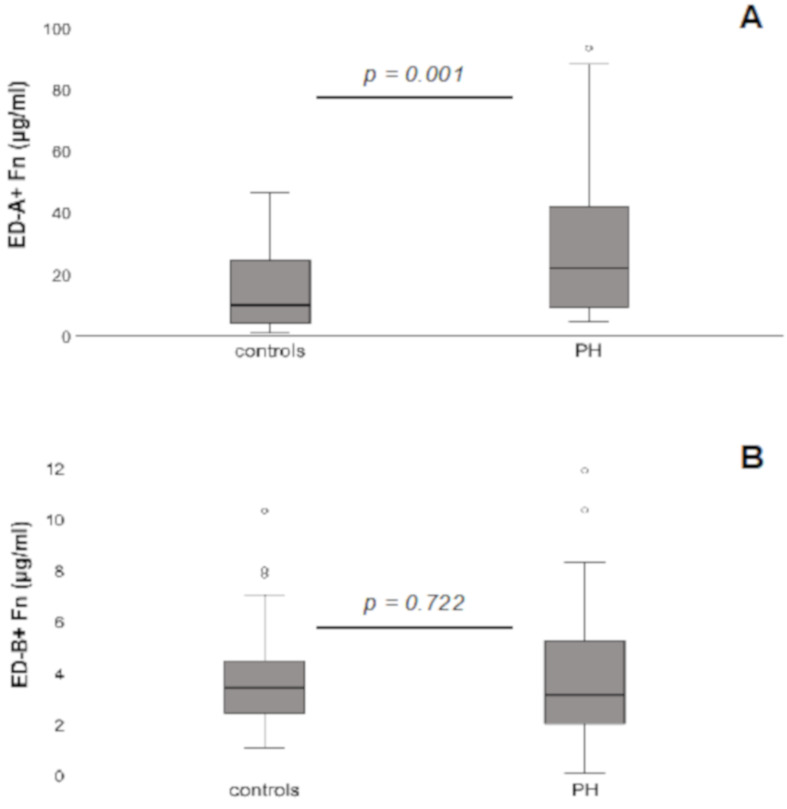
Increased serum levels of ED-A^+^ Fn (**A**) but not ED-B^+^ Fn (**B**) in patients with pulmonary hypertension (PH) compared to healthy controls. The box plots indicate the median (line inside the box), 25th and 75th percentile (lower and upper boundary of the box), 10th and 90th percentile (whiskers outside the box), as well as outlier values (dots). Fn = Fibronectin.

Quantification of the serum concentrations of ED-A^+^ Fn revealed significantly higher levels in PH patients (22.2 ± 23.3 μg/mL) compared to controls (9.9 ± 13 μg/mL; *p* = 0.001; Figure 1A); for ED-B^+^ Fn, no significant differences could be detected (PH patients: 3.1 ± 2.4 μg/mL; controls: 3.4 ± 2.2 μg/mL; *p* = 0.722; Figure 1B). Since there were no differences for ED-B^+^ Fn, the following analyses were performed only for ED-A^+^ Fn.

### 3.3. Serum Levels of ED-A^+^ Fn in Comparison between the Different Aetiological Groups within the PH Patients’ Collective Compared to Controls

**Figure 2:**
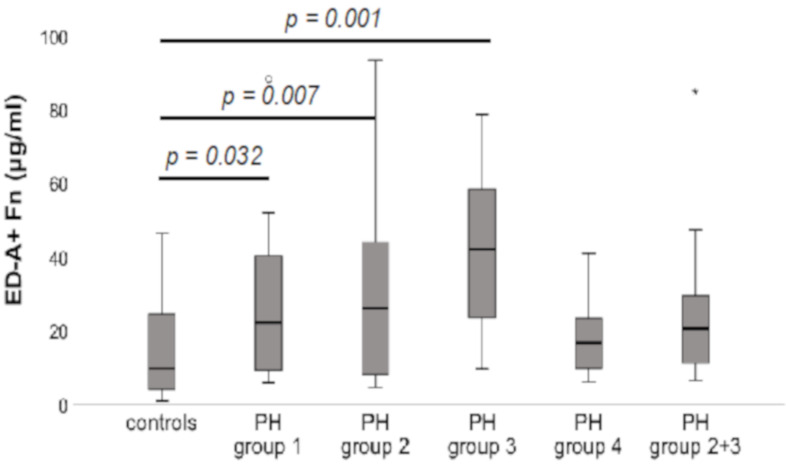
Subgroup analysis of the ED-A^+^ Fn serum levels in patients with pulmonary hypertension (PH) compared to healthy controls. Subgroup analyses were performed according to the different clinical PH groups 1–4 according to current guidelines. The box plots indicate the median (line inside the box), 25th and 75th percentile (lower and upper boundary of the box), 10th and 90th percentile (whiskers outside the box), as well as outlier values (dots). PH = pulmonary hypertension, Fn = Fibronectin.

When compared to controls (9.9 ± 13 μg/mL), significantly increased serum levels of ED-A^+^ Fn could be detected for the PH groups 1 (22.2 ± 24.3 μg/mL; *p* = 0.032), 2 (26.1 ± 26.7 μg/mL; *p* = 0.007) and 3 (42.2 ± 22.2 μg/mL; *p* = 0.001). In contrast, for group 4 (16.8 ± 11.1 μg/mL) as well as the combined group 2/3 (20.7 ± 21.2 μg/mL), the serum concentrations did not significantly differ from the controls (*p* = n.s.). There were no significant differences in ED-A^+^ Fn levels between the groups 1, 2 and 3 (*p* = n.s.). The results are shown in Figure 2.

### 3.4. Correlation Analyses of ED-A^+^ Fn Serum Levels with Brain Natriuretic Peptide, 6-Minute Walk Distance and Echocardiographic Parameters

**Figure 3:**
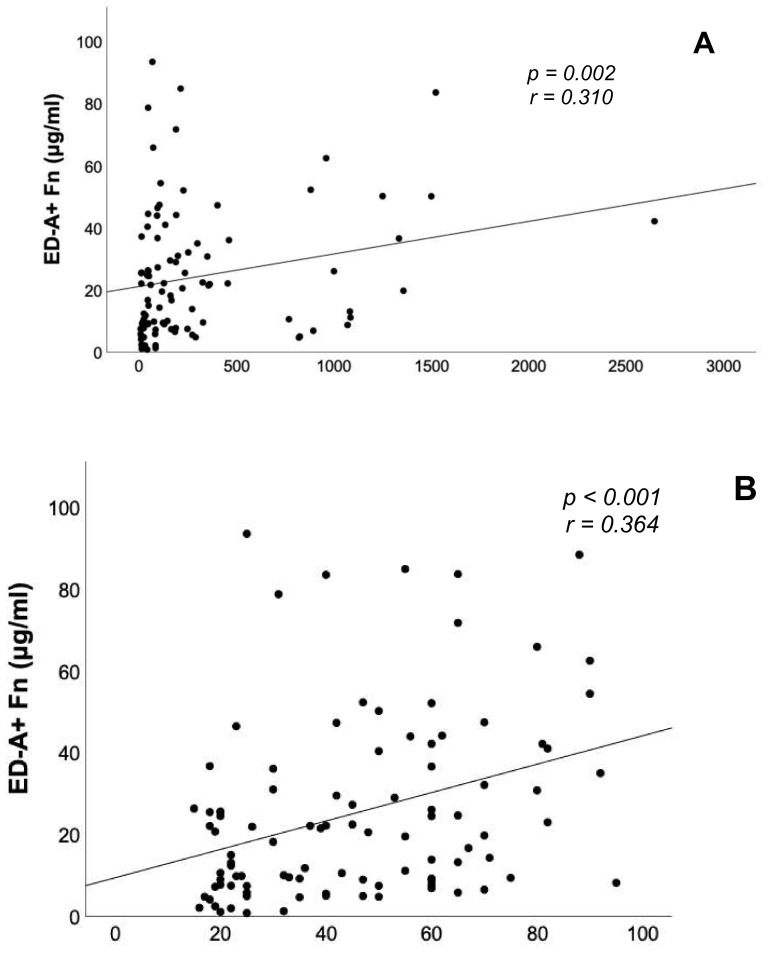

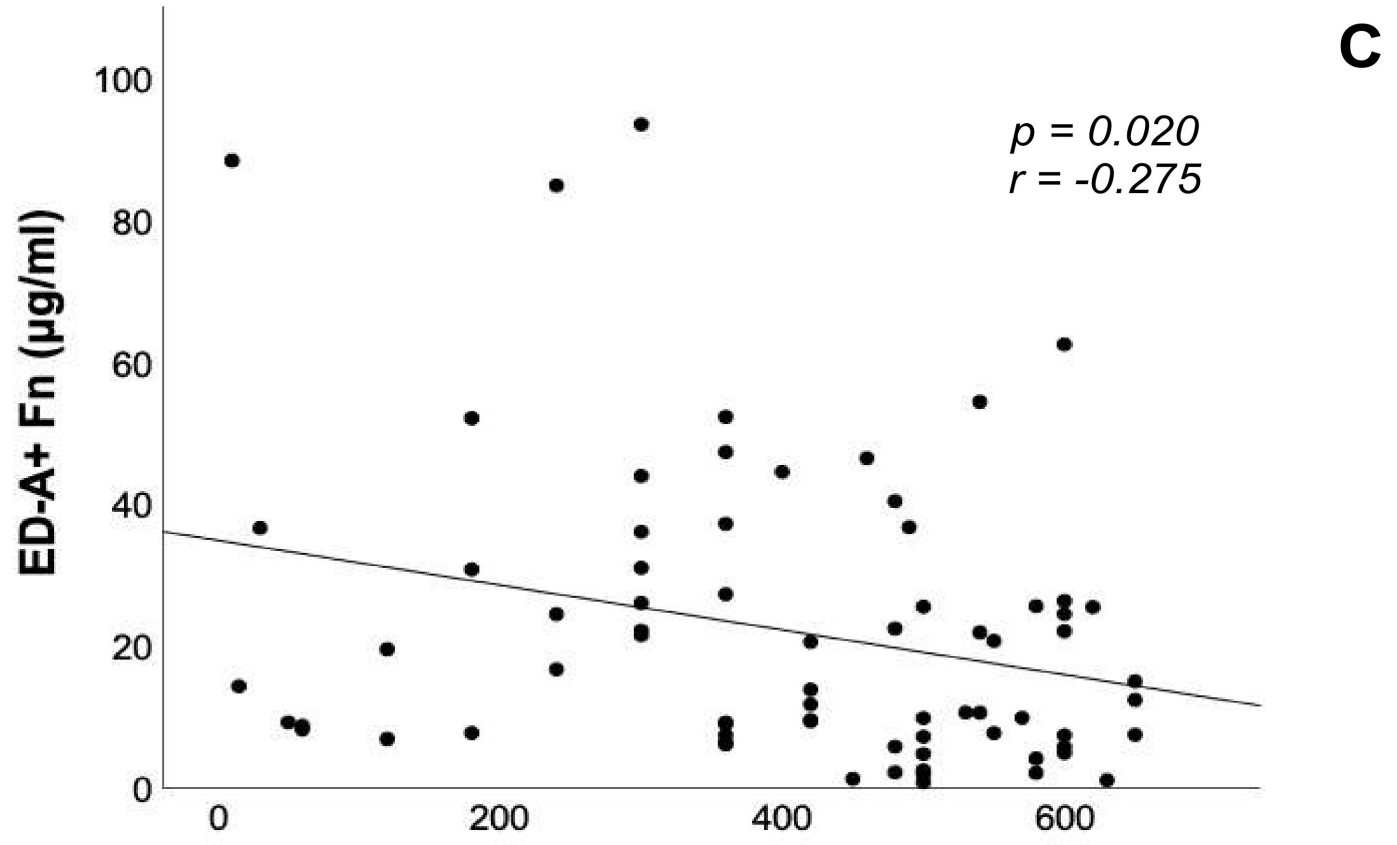
Correlation analyses between the serum concentration of ED-A^+^ Fn and serum brain natriuretic peptide levels, 6-minute walk distance as well as systolic pulmonary artery pressure assessed by echocardiography. The correlation analysis graphs demonstrate significant correlations between ED-A^+^ Fn and serum BNP levels (**A**), PAPsys (**B**) as well as SMWD (**C**). BNP: brain natriuretic peptide, PAPsys: systolic pulmonary arterial pressure, SMWD: 6-minute walk distance, Fn: fibronectin, *p*-value: level of significance, r-value: correlation coefficient.

Within the study collective, there were significant positive correlations between the serum concentrations of ED-A^+^ Fn and BNP levels (*r* = 0.310, *p* = 0.002, Figure 3A) as well as the PAPsys assessed by TTE (*r* = 0.364, *p* < 0.001, Figure 3B). In addition, a significant inverse correlation could be evidenced between ED-A^+^ Fn serum levels and SMWD (*r* = −0.275, *p* = 0.020, Figure 3C). For the remaining echocardiographic parameters assessed in this study, e.g., right ventricular diameters and function or right atrial area, no significant correlations with ED-A^+^ Fn serum levels were observed. With respect to the invasively assessed hemodynamic parameters (Table 3), there was a significant positive correlation between ED-A^+^ Fn and CI (*r* = 0.392, *p* = 0.002).

### 3.5. Multivariate Analysis and ROC Curves

To test the value of serum ED-A^+^ Fn level as a predictor for the presence of PH, a multivariate analysis was performed as described in material and methods. Age, diabetes mellitus, coronary artery disease, atrial fibrillation, chronic kidney disease, C-reactive protein, statin therapy, chronic obstructive pulmonary disease, arterial hypertension and ED-A^+^ Fn were entered into the analysis as independent variables. After multistep backward elimination, out of the 10 variables included, only chronic kidney disease (Wald: 7.090, OR: 8.866, 95%CI: 1.779–44.187, *p* = 0.008), C-reactive protein (Wald: 4.347, OR: 1.194, 95%CI: 1.011–1.410, *p* = 0.037) and ED-A^+^ Fn (Wald: 6.757, OR: 1.045, 95%CI: 1.011–1.080, *p* = 0.009) could be evidenced to be independent predictors of PH. Thus, a step-up of 1.0μg/mL in serum concentration of ED-A^+^ Fn is accompanied by a 4.5% increased probability for the presence of PH.

**Figure 4:**
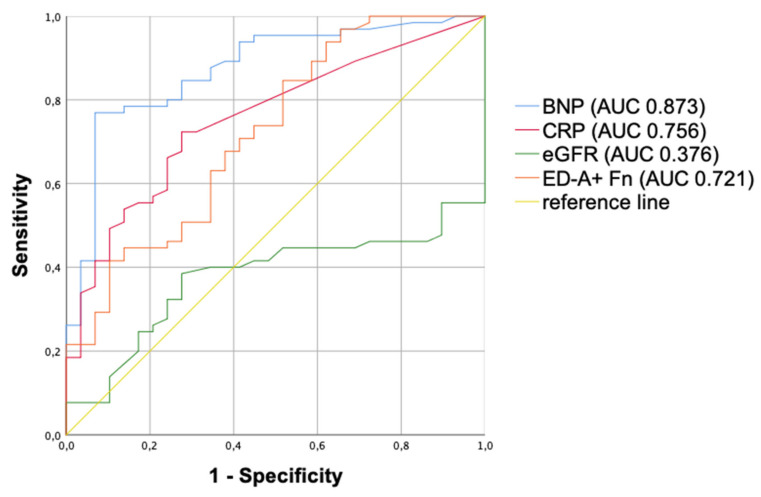
ROC curves to distinguish between PH patients and healthy controls, including AUC values for ED-A^+^ Fn, BNP, CRP and eGFR. ROC: receiver operating characteristic, BNP: brain natriuretic peptide, CRP: C reactive protein, eGFR: estimated glomerular filtration rate, PH: pulmonary hypertension, AUC: area under the curve, *p*-value: level of significance.

Moreover, based on these results and the strong correlation between ED-A^+^ Fn and BNP, ROC curves to distinguish PH patients from healthy controls were performed for ED-A^+^ Fn, BNP, CRP and eGFR (Figure 4).

## 4. Discussion

For the management of PH as a clinical and aetiological heterogeneous disease group, there are unmet clinical needs in terms of the establishment of novel biomarkers or even therapeutic targets. Pulmonary vascular and consecutively developing right ventricular myocardial tissue remodelling is associated with a comparable structural and functional reorganization of the cardiovascular ECM, at least for most aetiologies.

Thus, analysis of the dynamic changes of its key components in the PH disease continuum might help to identify molecules of interest possibly contributing to improved clinical management. In particular, the disease-associated re-expression of fetal Fn variants is of certain interest since these molecules are not only deposited into tissue but also liberated into the circulation [20,27,32,33].

In the current study, we quantified serum levels of both ED-A^+^ and ED-B^+^ Fn in the serum of PH patients belonging to different clinical groups in comparison to healthy controls using non-commercially available ELISA protocols developed in our group ^33^. When comparing ED-A^+^ and ED-B^+^ Fn serum levels in PH patients and healthy controls, we observed significantly increased levels for ED-A^+^ but not for ED-B^+^ Fn qualifying ED-A^+^ Fn as a promising biomarker in PH. This goes in line with recent findings of our group showing a strong re-expression of the molecule in human tissue and serum samples from patients suffering from, e.g., coronary artery disease, aortic stenosis, heart failure of different aetiology or rejection after heart transplantation [26,27,28,30,33,36]. Moreover, we could report similar findings in several preclinical models of cardiac allograft vasculopathy or PH, respectively [29,31,32,37]. In a very recent study, including elderly patients suffering from severe symptomatic aortic stenosis, our group investigated a panel of biomarkers with respect to their association to the novel staging classification of extra-valvular cardiac damage [38]. Interestingly, ED-A^+^ Fn was significantly increased in aortic stenosis patients additionally exhibiting PH with or without consecutive right heart failure, compared to patients in which the disease was restricted to the left side of the heart [36]. In contrast to ED-A^+^ Fn, a variety of studies investigating ED-B^+^ Fn revealed that its re-expression is strongly linked to cancer development and tumour-associated angiogenesis and not to non-neoplastic chronic inflammatory diseases [39,40,41]. These data might explain our current finding concerning ED-B^+^ Fn, which did not show any relevant alterations in the serum of patients suffering from PH [11].

Interestingly, when looking into the different PH groups according to current guidelines [1,2], we found significantly increased serum levels of ED-A^+^ Fn in PH group 1, 2 and 3, each compared to healthy controls, not being present in PH group 4. This finding might be supported by the fact that histopathological remodelling processes are more or less unique in PH groups 1, 2 and 3 but present in a different way in PH group 4, which is chronic thromboembolic pulmonary hypertension (CTEPH) [13,14,15,42]. The latter phenomenon seems to be comprehensible when considering pathogenesis of PH group 4 as a consequence of a chronic vessel occlusion due to recurrent pulmonary arterial embolisms and not caused by pulmonary vascular remodelling triggered by aetiology-dependent mechanisms as described for groups 1, 2 and 3 [11].

When further interpreting our results, the question arises, whether exclusively the pulmonary vasculature or also the consecutively affected right ventricle is the primary source of circulating ED-A^+^ Fn. From our current data, we cannot state this interesting point concludingly. However, in recent studies of our group, we could show in preclinical animal models of PH that ED-A^+^ Fn is re-expressed both in the pulmonary vasculature as well as in the remodelled right ventricle [31,32].

To further test the value of ED-A^+^ Fn as a biomarker improving PH diagnosis, we correlated the serum concentrations to common clinical, laboratory, functional and echocardiographic parameters. Most notably, we could demonstrate a strong positive correlation of ED-A^+^ Fn to BNP serum levels and systolic PAP assessed by echocardiography. Furthermore, there was a strong negative correlation between ED-A^+^ Fn and SMWD.

The association to BNP serum levels, likely originating from the right ventricle affected by pressure overload, is of certain interest since a re-occurrence of ED-A^+^ Fn in the process of cardiac tissue remodelling has been described in several studies of our group, both in tissue and in the circulation [27,28,30,33]. In that context, one could speculate that also ED-A^+^ Fn might, at least in part, originate from the right ventricular myocardium, as already discussed above.

The relation between increased ED-A^+^ Fn serum levels and an elevated PAP has been shown in a rat model of MCT-induced PH by our group recently [32]. Since both increased PAP as well as associated right heart failure are of prognostic relevance in PH [4,5], measurement of ED-A^+^ Fn and investigation of its relation to outcome parameters and mortality should be the object of future clinical trials including large patient numbers.

Additionally, the negative association of ED-A^+^ Fn serum levels and SMWD as a well-established parameter of functional performance recommended in current PH guidelines could be an impetus for the further evaluation of the molecule as a therapy surveillance marker, especially in PH group 1 (PAH) patients receiving a specific therapy [7].

In a multivariate analysis, we could identify ED-A^+^ Fn serum levels together with CKD and CRP levels as independent predictors of PH. In ROC curve analysis to distinguish between PH patients and healthy controls, AUC values were comparable between BNP, CRP and ED-A^+^ Fn. The validity of this interesting finding is limited by the low number of patients included in our current preliminary study but could motivate further testing of ED-A^+^ Fn also as a screening marker of PH. This would be of certain clinical interest since reliable serum biomarkers for disease screening do not exist until now. Especially when considering the fact that PH is often diagnosed much too late, in functional classes III and IV being connected with very poor prognosis [43,44], laboratory screening tests could significantly improve patients’ outcomes.

## 5. Limitations

The disease-associated re-occurrence of ED-A^+^ Fn is not restricted to PH and subsequent right heart failure but is also observable in some other cardiovascular or chronic inflammatory disorders. Nevertheless, this particular splicing variant of Fn is nearly absent in healthy adult organs and becomes re-expressed, particularly in vascular remodelling and fibrosis development. In that context, a crucial role of ED-A^+^ Fn has been described, among others, in atherosclerosis, cardiac allograft vasculopathy and fibrosis, as well as ischemic cardiomyopathy [26,28,29,33,45]. Hence, the authors cannot exclude that ED-A^+^ Fn originates not exclusively from pulmonary vascular and right ventricular myocardial remodelling in PH but also, at least in part, from the underlying disease, especially in PH groups 2 and 3.

## 6. Conclusions

Taken together, in this preliminary study, we could show that ED-A^+^ but not ED-B^+^ Fn seems to be a promising novel biomarker in PH, especially in the clinical groups 1, 2 and 3. The potential value as a novel molecular target for, e.g., antibody-based targeted delivery of bioactive payloads or even in terms of functional blocking of the molecule itself, should be the object of further preclinical studies, which are currently performed by our group.

## Figures and Tables

**Table 1:** Characteristics of the study groups in comparison between the PH patients in the total (*n* = 80) and controls (*n* = 40).

	Controls (*n* = 40)	PH Patients (*n* = 80)	*p*-Value
**Clinical Parameters**			
Age (years)	66 ± 7	71 ± 13	<0.001
Male gender (%)	33	39	n.s.
BMI (kg/m^2^)	27.9 ± 4.7	28.6 ± 6.0	n.s.
Systolic BP (mmHg)	146 ± 31	143 ± 25	n.s.
Diastolic BP (mmHg)	81 ± 16	81 ± 14	n.s.
Functional class	1.5 ± 0.6	2.6 ± 0.8	<0.001
**Comorbidities**			
Arterial hypertension (%)	95	83	n.s.
Coronary artery disease/infarction (%)	0	26	0.001
Hypertensive heart disease (%)	59	50	n.s.
Atrial fibrillation (%)	18	49	0.002
Pulmonary diseases (%)	5	44	<0.001
COPD (%)	3	24	0.004
Pulmonary fibrosis (%)	0	11	0.031
Chronic kidney disease (%), viz. GFR < 50 mL/min	8	49	<0.001
Hyperlipidaemia (%)	85	57	0.002
Diabetes mellitus (%)	20	49	0.002
Obesity (%), viz. BMI > 30 kg/m^2^	43	38	n.s.
Autoimmune diseases (%)	0	16	0.009
Smoking (%)	29	52	n.s.
**Medication**			
ASA (%)	25	19	n.s.
Beta blockers (%)	63	64	n.s.
ACE-Inhibitors/Sartans (%)	85	76	n.s.
Calcium channel blockers (%)	25	27	n.s.
Diuretics (%)	45	80	<0.001
Statins (%)	38	60	0.022
Prednisolone (%)	0	12	0.025
Inhaled Corticosteroids (%)	5	23	0.016
**Laboratory**			
BNP (pg/mL)	34 ± 70	190 ± 584	<0.001
CRP (mg/L)	2.0 ± 2.6	5.0 ± 17.5	<0.001
Creatinine (µmol/L)	71 ± 16	100 ± 50	<0.001
LDL (mmol/L)	3.5 ± 1,0	2.6 ± 1.0	<0.001
Haemoglobin (mmol/L)	8.8 ± 0,8	8.0 ± 1.4	0.001
Leukocytes (Gpt/L)	7.0 ± 1.4	7.2 ± 2.2	n.s.
Platelets (Gpt/L)	237 ± 48	232 ± 76	n.s.

Data presented as mean ± standard deviation or percentage. Laboratory parameters represented as median ± standard deviation. Abbreviations: ACE: Angiotensin-converting enzyme, ASA: acetylsalicylic acid, BMI: Body Mass Index, BNP: brain natriuretic peptide, BP = blood pressure, COPD: chronic obstructive pulmonary disease, CRP: C-reactive protein, GFR: glomerular filtration rate, LDL: low-density lipoprotein, n: number, n.s.: not significant, PH: pulmonary hypertension.

**Table 2:** Characteristics of the study groups in comparison between PH patient subgroups and controls.

	Controls	PH Group 1	PH Group 2	PH Group 3	PH Group 4	PH Group 2 + 3	*p*-Value between Different PH Groups
*n*	40	13	30	11	12	14	
**Clinical parameters**							
Age (years)	66 ± 7	66 ± 12	76 ± 8	59 ± 22	71 ± 12	76 ± 8	0.015
Male gender (%)	33	15	37	45	50	50	n.s.
BMI (kg/m^2^)	27.9 ± 4.7	29.0 ± 8.4	27.9 ± 3.9	25.9 ± 7.5	30.6 ± 5.2	29.9 ± 6.5	n.s.
Systolic BP (mmHg)	146 ± 31	134 ± 17	156 ± 31	142 ± 22	143 ± 20	135 ± 26	n.s.
Diastolic BP (mmHg)	81 ± 16	78 ± 8	80 ± 14	85 ± 9	83 ± 16	79 ± 20	n.s.
Functional class	1.5 ± 0.6	2.6 ± 0.8	2.6 ± 0.7	2.4 ± 1.1	2.3 ± 0.7	2.8 ± 0.7	n.s.
**Comorbidities**							
Arterial Hypertension (%)	95	77	93	70	83	77	n.s.
Coronary artery disease/infarction (%)	0	15	33	36	0	36	n.s.
Hypertensive heart disease (%)	59	27	70	27	17	71	0.002
Atrial fibrillation (%)	18	8	73	45	17	64	<0.001
Pulmonary diseases (%)	5	54	10	91	25	86	<0.001
COPD (%)	3	8	10	50	17	57	0.002
Pulmonary fibrosis (%)	0	15	0	20	0	36	0.006
Chronic kidney disease (%), GFR < 50 mL/min	8	23	59	36	36	71	n.s.
Hyperlipidaemia (%)	85	36	57	60	75	54	n.s.
Diabetes mellitus (%)	20	31	60	90	25	36	0.009
Obesity (%), BMI > 30 kg/m^2^	43	40	30	33	36	58	n.s.
Autoimmune diseases (%)	0	38	7	27	0	21	0.037
Smoking (%)	29	20	63	33	60	67	n.s.
**Medication**							
ASA (%)	25	17	21	20	0	36	n.s.
Beta blockers (%)	63	38	90	40	42	73	0.002
ACE-Inhibitors/Sartans (%)	85	62	86	60	75	82	n.s.
Calcium channel blockers (%)	25	31	28	30	8	36	n.s.
Diuretics (%)	45	92	83	60	67	91	n.s.
Statins (%)	38	54	76	40	50	55	n.s.
Prednisolone (%)	0	15	3	40	0	15	0.022
Inhaled Corticosteroids (%)	5	38	7	50	8	36	0.013
**Laboratory**							
BNP (pg/ml)	34 ± 70	104 ± 82	325 ± 678	326 ± 469	117 ± 87	285 ± 731	<0.001
CRP (mg/L)	2.0 ± 2.6	3.7 ± 10.7	5.5 ± 21.0	7.6 ± 12.9	2.6 ± 5.8	8.4 ± 22.0	n.s.
Creatinine (µmol/L)	71 ± 16	70 ± 44	123 ± 59	100 ± 24	86 ± 40	103 ± 47	0.019
LDL (mmol/L)	3.5 ± 1.0	2.4 ± 1.0	2.4 ± 1.0	2.9 ± 1.0	2.9 ± 0.9	2.8 ± 1.2	n.s.
Haemoglobin (mmol/L)	8.8 ± 0.8	8.0 ± 1.5	7.3 ± 1.2	8.4 ±1.0	9.5 ± 1.4	7.9 ± 1.6	0.014
Leukocytes (Gpt/L)	7.0 ± 1.4	6.0 ± 2.4	7.6 ± 1.7	7.7 ±1.6	7.4 ± 1.8	6.6 ± 3.4	n.s.
Platelets (Gpt/L)	237 ± 48	232 ± 51	224 ± 82	303 ±63	297 ± 73	193 ± 70	0.012

Data presented as mean ± standard deviation or percentage. Laboratory parameters represented as median ± standard deviation. Abbreviations: ACE: Angiotensin-converting enzyme, ASA: acetylsalicylic acid, BMI: Body Mass Index, BNP: brain natriuretic peptide, BP: blood pressure, COPD: chronic obstructive pulmonary disease, CRP: C-reactive protein, GFR: glomerular filtration rate, LDL: low-density lipoprotein, n: number, n.s.: not significant, PH: pulmonary hypertension.

**Table 3:** Hemodynamic parameters of the different PH groups as well as the subgroups of PH group 1.

Parameter	PH Group 1 (*n* = 13)	IPAH (*n* = 7)	CTD-PAH (*n* = 4)	CHD-PAH/Heritable (*n* = 2)	PH Group 2 (*n* = 30)	PH Group 3 (*n* = 11)	PH Group 4 (*n* = 12)	PH Group 2 + 3 (*n* = 14)	*p* Value *
mPAP (mmHg)	53 ± 12	55 ± 14	53 ± 12	45 ± 1	39 ± 11	37 ± 9	43 ± 16	45 ± 10	0.021
PCWP (mmHg)	12 ± 3	12 ± 2	13 ± 5	11 ± 5	23 ± 6	12 ± 11	14 ± 5	24 ± 8	<0.001
PVR (dyn*sec*cm^−5^)	755 ± 412	799 ± 406	781 ± 541	548 ± 252	321 ± 191	518 ± 287	568 ± 447	352 ± 249	0.002
mRAP (mmHg)	9 ± 7	11 ± 5	9 ± 8	N/A	12 ± 5	6 ± 4	10 ± 6	15 ± 8	0.017
CI ((L/min)/m^2^)	2.5 ± 0.6	2.6 ± 0.7	2.1 ± 0.3	3 ± 0.7	2.2 ± 0.5	2.2 ± 0.7	2.0 ± 0.3	2.4 ± 0.8	n.s.

Data presented as mean ± standard deviation. * *p* value between the different PH groups. Abbreviations: mPAP: mean pulmonary artery pressure, PCWP: pulmonary capillary wedge pressure, PVR: pulmonary vascular resistance, mRAP: mean right atrial pressure, CI: cardiac index, PH: pulmonary hypertension.

**Table 4:** Echocardiographic parameters of the study groups in comparison between PH patient subgroups (PH groups) and controls.

Parameter	Controls	PH Patients	*p* Value *	PH Group 1	PH Group 2	PH Group 3	PH Group 4	PH Group 2 + 3	*p* Value **
PAP_sys_ (mmHg)	21 ± 4	55 ± 18	<0,001	59 ± 22	52 ± 16	57 ± 22	50 ± 20	56 ± 16	n.s.
RA area (cm^2^)	15 ± 2	25 ± 10	<0.001	21 ± 8	28 ± 10	20 ± 9	22 ± 5	31 ± 14	n.s.
RVEDd (mm)	35 ± 2	45 ± 8	<0.001	41 ± 9	46 ± 7	47 ± 10	44 ± 7	49 ± 9	n.s.
LVEF (%)	68 ± 7	58 ± 11	<0.001	63 ± 7	56 ± 13	63 ± 9	60 ± 6	52 ± 12	0.050

Data presented as mean ± standard deviation. * *p* value between controls and PH patients; ** *p* value between the different PH groups. Abbreviations: PAP_sys_: systolic pulmonary artery pressure, RA area: right atrial area, RVEDd: right ventricular end-diastolic diameter, LVEF: left ventricular ejection fraction, PH: pulmonary hypertension.

## Data Availability

The data presented in this study are available in this manuscript.
